# Sea-Ice Bacteria *Halomonas* sp. Strain 363 and *Paracoccus* sp. Strain 392 Produce Multiple Types of Poly-3-Hydroxyalkaonoic Acid (PHA) Storage Polymers at Low Temperature

**DOI:** 10.1128/AEM.00929-21

**Published:** 2021-08-11

**Authors:** E. Eronen-Rasimus, J. Hultman, T. Hai, I. S. Pessi, E. Collins, S. Wright, P. Laine, S. Viitamäki, C. Lyra, D. N. Thomas, P. N. Golyshin, A.-M. Luhtanen, H. Kuosa, H. Kaartokallio

**Affiliations:** a University of Helsinki, Faculty of Agriculture and Forestry, Department of Microbiology, Helsinki, Finland; b Finnish Environment Institute (SYKE), Marine Research Centre, Helsinki, Finland; c Bangor University, School of Natural Sciences, Bangor, United Kingdom; d University of Manitoba, Centre for Earth Observation Science, Winnipeg, Canada; e University of Helsinki, Institute of Biotechnology, Helsinki, Finland; f University of Helsinki, Faculty of Biological and Environmental Sciences, Ecosystems and Environment Research Programme, Helsinki, Finland; g University of Helsinki, Faculty of Biological and Environmental Sciences, Molecular and Integrative Biosciences Research Programme, Helsinki, Finland; North Carolina State University

**Keywords:** *Halomonas*, *Paracoccus*, poly-3-hydroxyalkanoic acid, PHA, SCL-PHA, MCL-PHA, copolymer, sea-ice bacteria, marine bacteria, transcriptomics, genomics

## Abstract

Poly-3-hydroxyalkanoic acids (PHAs) are bacterial storage polymers commonly used in bioplastic production. Halophilic bacteria are industrially interesting organisms, as their salinity tolerance and psychrophilic nature lowers sterility requirements and subsequent production costs. We investigated PHA synthesis in two bacterial strains, *Halomonas* sp. 363 and *Paracoccus* sp. 392, isolated from Southern Ocean sea ice and elucidated the related PHA biopolymer accumulation and composition with various approaches, such as transcriptomics, microscopy, and chromatography. We show that both bacterial strains produce PHAs at 4°C when the availability of nitrogen and/or oxygen limited growth. The genome of *Halomonas* sp. 363 carries three *phaC* synthase genes and transcribes genes along three PHA pathways (I to III), whereas *Paracoccus* sp. 392 carries only one *phaC* gene and transcribes genes along one pathway (I). Thus, *Halomonas* sp. 363 has a versatile repertoire of *phaC* genes and pathways enabling production of both short- and medium-chain-length PHA products.

**IMPORTANCE** Plastic pollution is one of the most topical threats to the health of the oceans and seas. One recognized way to alleviate the problem is to use degradable bioplastic materials in high-risk applications. PHA is a promising bioplastic material as it is nontoxic and fully produced and degraded by bacteria. Sea ice is an interesting environment for prospecting novel PHA-producing organisms, since traits advantageous to lower production costs, such as tolerance for high salinities and low temperatures, are common. We show that two sea-ice bacteria, *Halomonas* sp. 363 and *Paracoccus* sp. 392, are able to produce various types of PHA from inexpensive carbon sources. *Halomonas* sp. 363 is an especially interesting PHA-producing organism, since it has three different synthesis pathways to produce both short- and medium-chain-length PHAs.

## INTRODUCTION

Poly-3-hydroxyalkanoic acids (PHAs), the most common bacterial storage polymers, can be utilized as renewable and biodegradable plastics (1). Industrially, the challenge is to produce PHAs from inexpensive, nonrelated carbon (C) skeletons structurally different from those of PHA C sources such as glucose, for which marine bacteria, including *Halomonas* spp., have shown considerable potential (2–7). Moreover, the recent focus on marine plastic pollution has given rise to an urgent need to develop sustainable alternatives for petrochemical plastics at competitive prices (8). PHA is one of the most promising alternative materials because it is biocompatible, i.e., nontoxic for living organisms, and bacteria are able to synthesize and degrade it completely with hydrolases and depolymerases (9–11). In particular, medium-chain-length (MCL) PHAs and copolymers are more flexible and easier to process, thus making them the polymers preferred for industrial applications (12).

Halophilic and psychrophilic bacteria display advantages as potential bioplatforms for PHA production because both high salinity tolerance and growth at low temperatures reduce the risk of contamination during cultivation and the associated production costs (6, 13–15). Recent studies have shown that sea-ice bacteria possess PHA granules and synthase genes (16, 17), suggesting that PHA production is ecologically relevant to microbial populations inhabiting sea ice. Thus, sea ice, known for rapidly fluctuating environmental conditions, including combined high salinities (up to 216‰ at –21°C [88]) and low temperatures (18), is a promising biome in which to prospect for new PHA-producing bacteria.

PHAs are linear polyesters that accumulate in hydrophobic cytoplasmic inclusion bodies that many bacteria use for C and energy storage (19–21). PHAs are ideal storage polymers; they are highly reduced and due to their low solubility have negligible effects on osmotic pressure regulation in the cell (19). PHAs also enhance survival during environmental stresses such as oxygen (O_2_) deficiency, UV radiation, salinity, and cold (21–26), all of which are encountered in sea ice (17). Environmental stressors cause oxidative stress in the bacteria, increasing the concentrations of reactive oxygen species (ROS) in cells (27). These can be further detoxified enzymatically with antioxidants such as superoxidase dismutase and catalase, some of which use NADP and NAD(P)H as cofactors (27). During O_2_ deficiency, PHA can act as a sink for reducing power, because the NAD(P)H produced in glucose catabolism cannot be oxidized, which leads to high NAD(P)H/NAD(P) ratios and channeling of NAD(P)H to NAD(P)H-dependent *phaB* and subsequent PHA production (19, 28–30). Therefore, PHAs are used by bacteria to maintain cellular redox balance by either synthesizing or depolymerizing PHA, i.e., storing or producing reduced equivalents (19, 21, 26, 28, 29, 31). Most commonly, PHAs are produced when nutrient availability is not balanced, e.g., when nitrogen (N) or phosphorus limits the growth but there is excess C available, leading to channeling of the surplus acetyl-coenzyme A (CoA) and NAD(P)H to PHA production (20, 32). Again, nutrient limitation is a well-recorded feature in sea-ice habitats (33).

We investigated the conditions and cellular basis for the PHA production in two bacterial strains newly isolated from Southern Ocean sea ice, *Halomonas* sp. 363 (*Gammaproteobacteria*) and *Paracoccus* sp. 392 (*Alphaproteobacteria*). We verified the PHA production using transcriptomes, microscopy, and gas chromatography-mass spectrometry (GC-MS). We show that these two sea-ice bacteria can produce various types of PHAs from inexpensive C sources under N limitation and also under colimitation of N and O_2_ at low temperature.

## RESULTS

The aim of the study was to investigate the conditions and cellular basis for PHA production in two bacterial strains isolated from Southern Ocean sea ice, *Halomonas* sp. 363 (*Gammaproteobacteria*) and *Paracoccus* sp. 392 (*Alphaproteobacteria*). Shaker flask batch-culture experiments were conducted with *Halomonas* sp. 363 and *Paracoccus* sp. 392 under both N-limited and N-replete conditions (Fig. S1 in the supplemental material).

### PHA genes.

**FIG 1:**
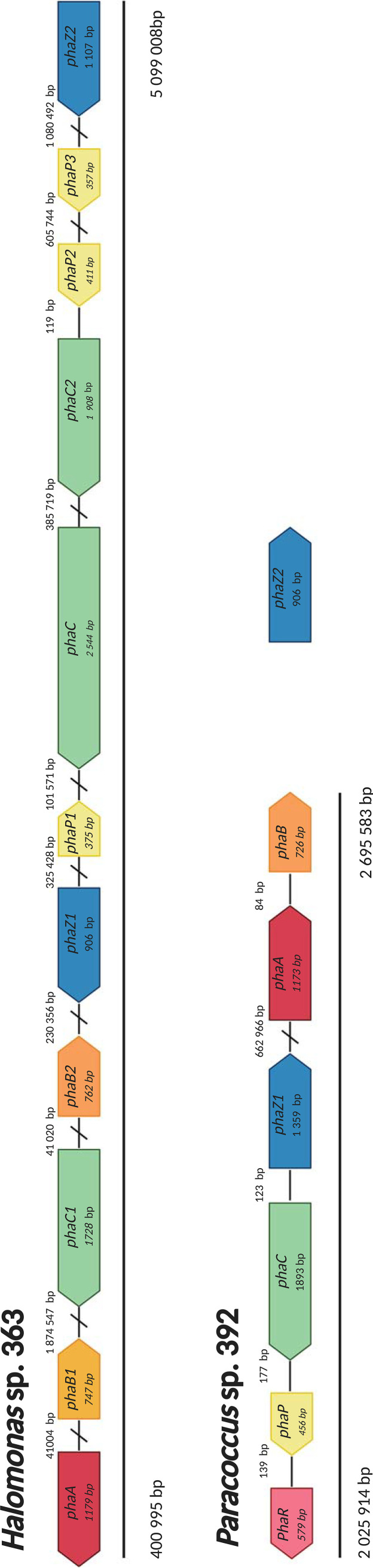
Annotated poly-3-hydroxyalkanoic acid (PHA) metabolic genes in the sea-ice bacteria *Halomonas* sp. 363 and *Paracoccus* sp. 392. The genome annotations against KEGG (release 86, April 2018) (80), PROKKA (1.13) (39), and RAST (2.0) (38) are listed in Tables S1 and S2.

**FIG 2:**
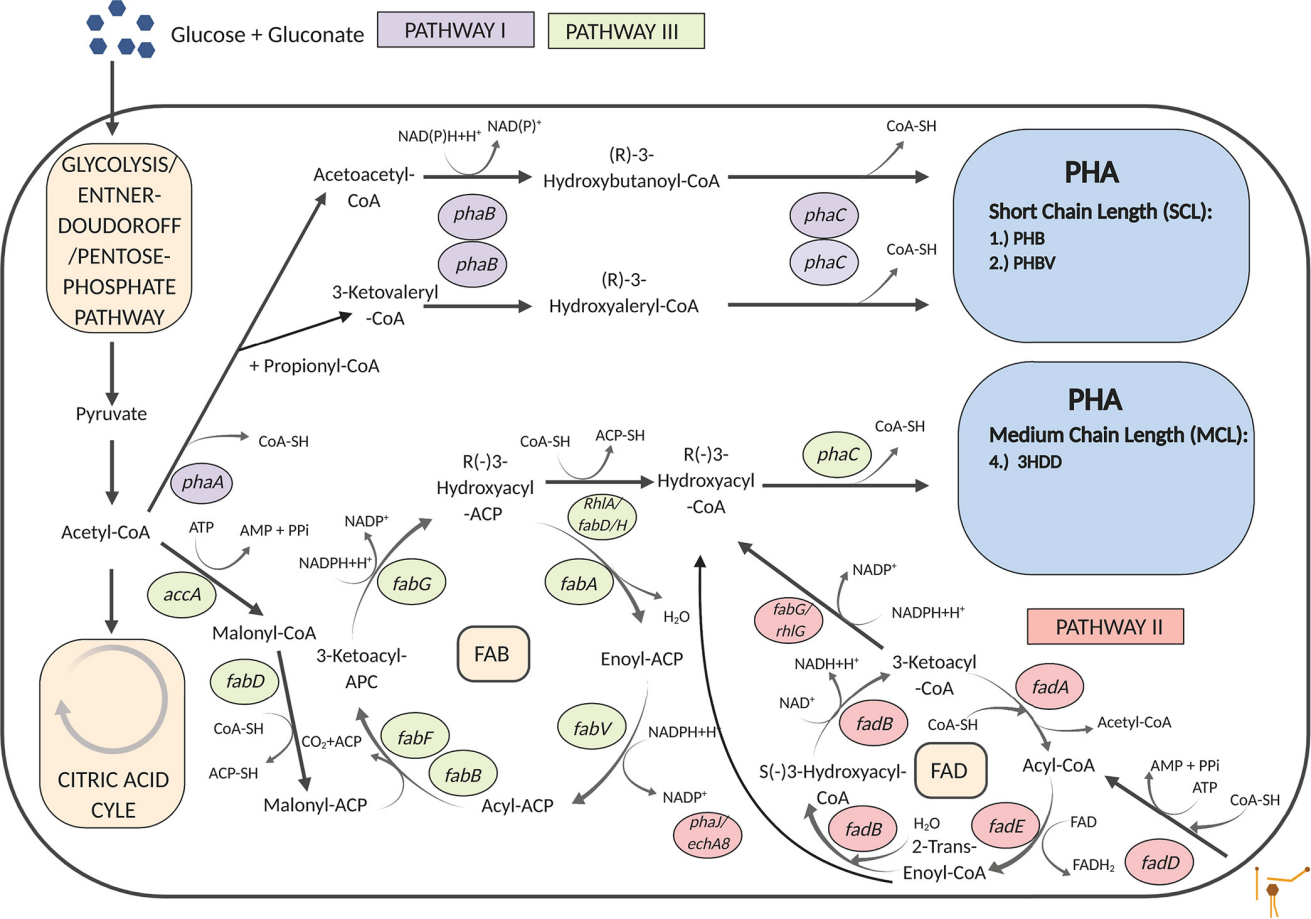
Actively transcribed genes putatively associated with poly-3-hydroxyalkanoic acid (PHA) synthesis in the sea-ice bacterial strains *Halomonas* sp. 363 and *Paracoccus* sp. 392. *Halomonas* sp. 363 has putatively three different pathways (pathways I to III) to produce both short-chain-length (SCL) and medium-chain-length (MCL) PHAs, whereas *Paracoccus* sp. 392 produces only SCL-PHA via pathway I. FAB, fatty-acid biosynthesis; FAD, fatty-acid degradation. The genes and their annotations are listed in Table S5. Numbering of the genes is not included in the schematic because we have not proven the pathways with knockout mutant strains.

The closed circular genome of *Halomonas* sp. 363 comprises 5.6 Mb and that of *Paracoccus* sp. 392 3.03 Mb along with 18 plasmids (range of plasmid length 0.003 to 0.33 Mb, complete genome 4.5 Mb). Both strains harbored all the genes (*phaA*, *phaB*, and *phaC*) essential for PHA production (Fig. 1, Fig. S2 and S3). In addition, both strains contained the phasin (*phaP*) and depolymerase (*phaZ*) genes, while *Paracoccus* sp. 392 also carried the regulator protein gene *phaR* (Fig. 2). One of the *Paracoccus* sp. 392 *phaZ* genes was carried by a plasmid (Fig. 1, Table S2). In *Halomonas* sp. 363, the PHA genes were scattered around the genome, as has been observed in other *Halomonas* strains (34–36), whereas in *Paracoccus* sp. 392 two gene clusters (*phaRPCZ* and *phaAB*) were identified (Fig. 1), in accordance with a previous study (37).

Based on annotations with RAST (38) and PROKKA (39), *Halomonas* sp. 363 harbors three *phaC* genes (*phaC*, *phaC1*, and *phaC2*); however, *phaC* was annotated only with RAST (Table S1). The predicted coding sequence (CDS) showed nonspecific matching with the class III PHA synthase (TIGR01836, 201 to 415 bp) based on the National Center for Biotechnology Information (NCBI) Conserved Domain Database (CDD). In addition, there was a stretch in the CDS (from amino acid [aa] 183 to 244; bp 549 to 732) which resulted in a 100% protein Basic Local Alignment Search Tool (BLASTp) hit against the nr database to the *phaC* gene in *Halomonas* (EHA17034.1). Moreover, the *phaC* gene was much larger (2,544 bp) than the synthase genes in general (1,622 to 1,973 bp) (40). Both exceptionally large *phaC* genes (7, 34) and strains with three *phaC* genes (41) have also been detected in other *Halomonas* strains. Since the *phaC* gene appears to be conserved in *Halomonas* spp. (Fig. S4), the results suggest it is a true gene.

In addition, *Halomonas* sp. 363 carries two copies of the *phaB* gene, as does the halophilic archaeon Haloferax mediterranei (42). This may have resulted from *Halomonas* having both an NADPH-dependent *phaB* gene for anabolic PHA production and another NADH-dependent *phaB* gene for PHA production under fermentative, O_2_-limited conditions, as suggested previously (30).

### Transcriptomes and PHA granule formation.

**FIG 3:**
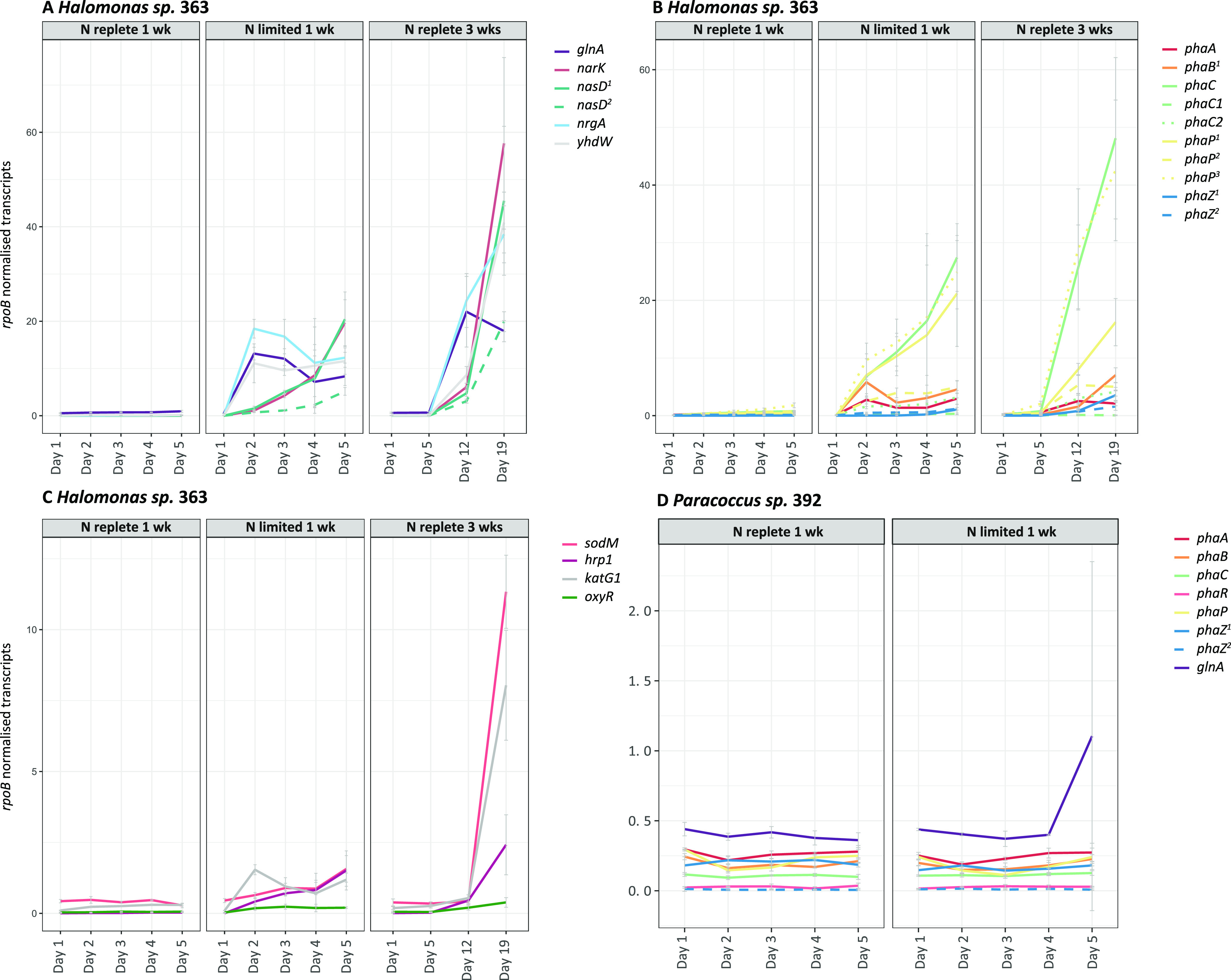
Actively transcribed genes from sea-ice bacterial strains *Halomonas* sp. 363 and *Paracoccus* sp. 392 are associated with the nitrogen cycle in *Halomonas* sp. 363 (A), poly-3-hydroxyalkanoic acid (PHA) production in *Halomonas* sp. 363 (B), oxygen limitation in *Halomonas* sp. 363 (C), and PHA production and nitrogen limitation in *Paracoccus* sp. 392 (D). The sequences were normalized against *rpoB*, after which the rRNA-associated genes were removed and the relative percentages counted. Note there are different scales on the different graphs. Complete transcriptome annotations against KEGG (release 86, April 2018) (80), PROKKA (1.13) (39), and RAST (2.0) (38) are listed in Tables S3 and S4.

In total, ∼834.2 million reads (∼173 Gb) were obtained with Nextseq. *Halomonas* sp. 363 contains transcribed genes for all three main PHA production pathways (I to III), of which the transcription level of pathway I was highest (Fig. 2 and 3D, Fig. S5, Table S3). *Paracoccus* sp. 392 contains transcribed genes only for pathway I (Fig. 2 and 3D, Table S4). In both strains, the PHA genes were transcribed in the N-limited 1-week treatments (Fig. 3B and D). By day 5, all *phaC* gene transcription levels increased significantly in *Halomonas* sp. 363 (one-way analysis of variance [ANOVA] *phaC P = *0.00142, F = 61.64; *phaC1 P* = 0.0157, F = 16.26; *phaC2 P = *0.018, F = 14.96) (Fig. S6A); however, the increase in *phaC1* and *phaC2* transcription was much lower than for *phaC*. No such increase was observed in *Paracoccus* sp. 392 (Fig. 3D and Fig. S6C). In addition, in *Halomonas* sp. 363, *phaC* and *PhaC2* gene transcription levels were significantly greater at the end of the N-replete 3-week treatment than on day 5 in the N-replete 1-week treatment (one-way ANOVA, *phaC P = *0.00424, F = 34.31; *phaC2 P = *0.00108, F = 71.22) (Fig. 3B and Fig. S6B), with the highest *phaC* activity observed throughout the experiment (Fig. 3B and 3D). However, it should be noted that the transcription level of the *phaC* gene in *Halomonas* sp. 363 was ∼10 times higher than *phaC1* and *phaC2* (Fig. 3B).

In the N-limited 1-week treatment in *Halomonas* sp. 363 from day 2 onward, and from day 12 onward in the N-replete 3-week treatment, the glutamine synthetase gene (*glnA*, NLJJMJOO_00241) was upregulated as an indicator of N deficiency, (Fig. 3A). The N limitation likely induced upregulation of the N uptake genes *nasD* (NLJJMJOO_01038 and NLJJMJOO_01039), *nrgA* (NLJJMJOO_04706), *yhdW* (NLJJMJOO_04689) (Fig. 3A and Fig. S2), and *narK* (NLJJMJOO_01066). The *narK* gene encodes a transporter responsible for nitrite/nitrate uptake across the cytoplasmic membrane, while *nasD* encodes a subunit of assimilatory nitrite reductase, *nrgA* an ammonium transporter, and *yhdW* an amino-acid transporter. The nutrient limitation appeared to be more severe in the N-limited 1-week treatment than in the N-replete 3-week treatment in *Halomonas* sp. 363, since the cells were larger in the latter, indicating that cells were not suffering from severe N limitation (Fig. S7C and D).

In *Paracoccus* sp. 392, the expression levels of *glnA* did not increase until day 4 in the N-limited 1-week treatment (Fig. 3D), indicating that *Paracoccus* sp. 392 likely used stored cellular N after transfer to the N-limited medium.

In addition to N limitation, the increases in the expression of superoxide dismutase (*sodM*), catalase-peroxidase 1 (*katG1*), activator for hydrogen peroxide-inducible genes (*oxyR*), and hypoxic response protein 1 (*hrp1*) genes (Fig. 3C) indicated O_2_ deficiency in the N-replete 3-week treatment in *Halomonas* sp. 363. Facultative anaerobes use superoxidase dismutase with catalase, or peroxidase, to protect anaerobic metabolism in the presence of O_2_ (43). A rapid increase in *phaC* expression coinciding with the upregulation of antioxidant and N limitation genes suggests that colimitation of N and O_2_ induced an increase in PHA production in the N-replete 3-week treatment. High cell densities combined with low rotation speed (120 rpm) led to microaerobic conditions and enhanced PHA accumulation in cultures (30, 44).

### PHA composition.

*Halomonas* sp. 363 produced mainly poly-3-hydroxybutyrate (PHB) (up to 45% [wt/wt]) (Table 1) from glucose and gluconate. Under N-limited conditions, trace amounts of beta-hydroxyvaleric (3-HV) and beta-hydroxydodecanoic (HDD) acid moieties were observed, although not quantified. Interestingly, the *Halomonas* sp. 363 N-replete 1-week treatment also resulted in accumulation of PHB (∼17% [wt/wt]) (Table 1). *Paracoccus* sp. 392 produced poly(3-hydroxybutyrate-co-3-hydroxyvalerate) (PHBV) copolymer with a range of 8.7% (wt/wt) 3-HB and 4.5% (wt/wt) 3-HV, while the N-replete treatment produced similar molarities of 3-HB and 3-HV (Table 1) from glucose and gluconate.

**TABLE 1:** Extracted PHA biopolyesters in *Halomonas* sp. 363 and *Paracoccus* sp. 392 cultured on glucose and gluconate under N-limited 1-week and N-replete 1-week conditions

Strains	Treatment	% dry matter^*a*^		
		% 3-HB	% 3-HV	% 3-HDD
*Halomonas* sp. 363	N-limiting 1-wk	45.00	traces	traces
	N-replete 1-wk	17.19	ND	ND
*Paracoccus* sp. 392	N-limiting 1-wk	8.71	4.51	ND
	N-replete 1-wk	8.52	4.17	ND

a PHA, poly-3-hydroxyalkanoic acid; 3-HB, 3-hydroxybutyrate; 3-HV, 3-hydroxyvalerate; 3-HDD, 3-hydroxydodecanoate; ND, not detected.

## DISCUSSION

PHAs are one of the most promising bioplastic materials, because they are fully synthesized and degraded by bacteria (11). We investigated PHA production and *pha* gene transcription in two bacterial strains, *Halomonas* sp. 363 and *Paracoccus* sp. 392, isolated from Southern Ocean sea ice, using shaker flask batch-culture experiments under N-limiting and N-replete growth conditions with glucose and gluconate as carbon sources. *Halomonas* sp. 363 produced mainly PHB, but trace amounts of PHBV and 3-hydroxydodecanoate (3-HDD) were also detected, whereas *Paracoccus* sp. 392 produced only PHBV. Since *Halomonas* sp. 363 tolerates high salinities and low temperatures and can exploit inexpensive carbon sources, as well as having three actively transcribed pathways (I to III) to produce PHAs with indications of MCL-PHA and copolymer production, *Halomonas* sp. 363 is an especially promising candidate for industrial PHA production.

### PHA genes and growth conditions.

PHA granules have a hydrophobic core, with amorphous PHA enclosed by a phospholipid layer that contains PHA synthase, depolymerase, phasin, and regulatory proteins embedded and attached (20, 40, 45). The key enzyme in PHA production is a synthase (PhaC) (40), which is divided into four classes (I to IV) based on the substrate specificity, subunit composition and sequence homology (10, 40). Class I, III, and IV synthases use short-chain-length (SCL) HA-CoAs (C_3_ to C_5_), whereas class II synthases use medium-chain-length (MCL) HA-CoAs (C_6_ to C_14_) as the substrates for polymerizing PHAs (40). *Halomonas* sp. 363 carries three *phaC* genes and produced SCL-PHA (PHB) in both N-limited and N-replete 1-week treatments, as well as in the N-replete 3-week treatment with combined N and O_2_ limitation. Based on microscopy and transcriptomes, the highest PHA yield was obtained under the combined N and O_2_ limitation, which occurred due to the low rotation speed of shaking flasks in an N-replete 3-week treatment. However, since the N-replete 3-week treatment was not analyzed with GC-MS, the result is based only on the observed higher transcription level of the *phaC* gene and visual inspection of micrographs.

In addition, trace amounts of MCL-PHA (3HDD) and copolymer PHBV were observed. MCL-PHA and copolymers are more flexible and have more desirable properties for industrial purposes, e.g., thermoplastic molding, compared with SCL-PHAs (12). Based on the MCL-PHAs detected, *Halomonas* sp. 363 apparently has synthase genes from different classes. Previously, *Halomonas* spp. *phaC* genes were regarded as class I, since they encode only enzymes producing SCL-PHAs and copolymers (6, 7, 36, 46–48), whereas MCL-PHAs are almost exclusively produced by Pseudomonas species or mutant strains (32). Interestingly, the Pseudomonas stutzeri *phaC2* gene product has very low substrate specificity and is capable of producing both SCL-PHAs and MCL-PHAs (3, 49, 50). In all, *Halomonas* sp. 363 appears to be the first wild-type strain that has been experimentally shown to possess the native capability for producing both SCL- and MCL-PHAs. However, further investigations are needed to directly link the genes to the PHA production observed and to determine the synthase class.

*Paracoccus* sp. 392 carries the class I *phaC* gene and produced small amounts of PHBV, both in the N-limited 1-week and N-replete 1-week treatments. However, based on *glnA* expression, N limitation was initiated only on day 5, likely explaining the small difference in PHA yield between the N-limited and N-replete treatments. Although bacteria more commonly produce PHA under nutrient-limiting conditions, these mechanisms vary, such that evidence shows bacteria can also produce PHA when nutrients are not exhausted (20, 32, 51). Another reason for the low PHBV concentration may be that the strains were cultured on glucose and gluconate and, for the valerate production, bacteria also need to use cell-derived substrates, such as amino acids, to produce the propionyl-CoA precursor (52). PHBV production in *Paracoccus* spp. has also been observed in previous studies (53, 54), although they are better known as a PHB producers (6, 37).

### PHA pathways.

PHAs are diverse and produced along several different pathways (I to VIII) from various C sources, including carbohydrates, amino acids, fatty acids, and CO_2_ (54–56). There are two main pathways from sugars; pathways I and III begin with acetyl-CoA as a precursor (56). In this study, glucose and gluconate were used as C sources to be processed along pathway I, producing SCL-PHAs and copolymers, and along the fatty-acid biosynthesis (FAB) pathway III, producing MCL-PHAs and copolymers (32, 51, 56–61). In *Halomonas* sp. 363, both pathway I and III genes were actively expressed, whereas in *Paracoccus* sp. 392 only pathway I genes were expressed. However, the transcription level of the pathway I genes in *Halomonas* sp. 363 was several times higher than for pathway III. Accordingly, *Halomonas* sp. 363 accumulated mostly SCL-PHA (PHB) but also showed indications of possible MCL-PHA (3HDD) and copolymer (PHBV) production, whereas *Paracoccus* sp. 392 accumulated only the PHBV copolymer (Table 1). The class II PHA synthases (pathway III) are capable of using exclusively CoA-linked 3-hydroxy acids (HAs), and thus a transacylating enzyme is needed to link FAB and PHA synthesis (57–59, 62). *phaG* catalyzes the conversion of (R)-3-hydroxyacyl-ACP to (R)-3-hydroxyacyl-CoA, which is further used as a substrate for *phaC* (57–59, 62). However, evidence is available that bacteria lacking the *phaG* gene, *rhlA* (63), and *fabD*, as well as *fabH* (64), may substitute to produce substrates for PHA synthase. In *Halomonas* sp. 363, all necessary genes for pathway III, except *phaG*, were annotated and expressed; however, it also carries *rhlA*, *fabD*, and *fabH* genes.

In addition to these two pathways, MCL-PHAs are produced from fatty acids along the fatty-acid degradation (FAD) pathway, i.e., pathway II (56). Interestingly, *Halomonas* sp. 363 also carries all the genes necessary for pathway II. Thus, *Halomonas* sp. 363 uses two fully annotated pathways to produce MCL-PHAs from both sugars and fatty acids. FAD genes have also been annotated from *Halomonas* sp. strain SF2003 (36). Since only trace amounts of 3HDD were detected in *Halomonas* sp. 363, it may be a product of pathway II derived from bacterial debris. Ecologically, the conversion of fatty acids to PHA likely occurs in sea ice, because sea-ice algae provide abundant fatty acids as bacterial C sources (18, 65).

In conclusion, PHA production was observed in the two Southern Ocean sea-ice bacteria *Halomonas* sp. 363 and *Paracoccus* sp. 392. Both strains produced PHAs from glucose and gluconate under N-limited and N-replete conditions at 4°C. Moreover, *Halomonas* sp. 363 also produced PHAs under combined N and O_2_ limitation. *Halomonas* sp. 363 is a particularly versatile organism with regard to PHA production, harboring genes for each of the three main pathways, as well as having the native capability of producing both SCL- and MCL-PHAs. In addition, it has several qualities that are considered industrially valuable for offsetting production costs, including the production of PHAs from inexpensive C sources under low aeration without compromising the cell size, as well as very flexible salinity and temperature tolerances.

## MATERIALS AND METHODS

### Bacterial strains.

Experiments were conducted with two Antarctic sea-ice bacteria, *Paracoccus* sp. 392 (*Alphaproteobacteria*) and *Halomonas* sp. 363 (*Gammaproteobacteria*) isolated from Southern Ocean sea ice (the isolation is described in reference 66). First, the strains were inoculated from a glycerol stock on modified ZoBell agar (5 g peptone, 1 g yeast extract, 15 g agar, 33 g Instant Ocean sea salt, 1,000 ml Milli-Q [MQ] water, autoclaved at 121°C for 20 min) (67). Single colonies were then inoculated into 50 ml of liquid ZoBell medium (5 g peptone, 1 g yeast extract, Instant Ocean sea salt, 1,000 ml MQ water, autoclaved at 121°C for 20 min) (67) for pregrowth at 4°C to a turbidity optical density (OD) (600 nm; bandwidth, 40 nm [Ultrospec 10; Biochrom Ltd, UK]) of 0.7 to 1.2 (3 days for *Halomonas* sp. 363; 6 days for *Paracoccus* sp. 392) in three replicates. The OD could not be measured reliably for *Paracoccus* sp. strain 392 because the bacterial cultures were too heterogeneous and organized in tight aggregates. From each culture, 1 ml of *Halomonas* sp. 363 and 8 ml of *Paracoccus* sp. 392 culture were inoculated into the N-replete experimental units (two from each; i.e., control and N-limitation treatment) for the phase I biomass accumulation (Fig. S1).

### Experimental setup.

PHA production was examined in 200-ml shaker flask batch cultures in the dark at 4°C on an orbital shaker set at 120 rpm, with three replicates for each treatment. The bacteria were cultured in two phases (Fig. S1): in phase I, six replicates from both strains were inoculated from the pregrowth medium to the 200-ml N-replete mineral media (MM; modified from reference 68) (Document S1 in the supplemental material). In phase I, the bacteria were cultured to achieve an OD of 0.7 to 1.2 on N-replete MM to accumulate biomass. In phase II, the cells were pelleted (13,000 × *g*, 3 min, 4°C); three pellets were inoculated to N-limited MM (modified from reference 68) (Document S1) to induce PHA production (N-limited 1-week treatment) and three to N-replete MM as a negative control (N-replete 1-week treatment). After the cells were collected and transferred to new medium (day 1), their growth was followed for 4 days and samples obtained daily for 5 days for transcriptomes (2 ml) and Nile blue microscopy (1 ml in 1.25% glutaraldehyde).

Surprisingly, *Halomonas* sp. 363 produced PHA under N-replete conditions, so an additional experiment (N-replete 3-week) was conducted to observe the effects of natural nutrient depletion on PHA production. Bacterial strains were prepared and cultured the same way as for the N-replete 1-week, but the cells were not pelleted or resuspended, and the incubation time was extended to 19 days. Samples were collected once per week for 3 weeks (days 1, 5, 12, and 19).

### Microscopy.

PHA production was verified microscopically. Samples for Nile blue staining were stored in electron microscopy-grade glutaraldehyde (final concentration of 1.25%) at 4°C. The Nile blue preparations were prepared as previously described (69). In short, 10 μl from the stock was pipetted onto microscopic slides, spread out, and dried for 15 min in a laminar-flow hood. The slides were flamed and immersed into preheated, 0.2-μm-filtered Nile blue solution for 10 min (water bath, 55°C). The slides were rinsed with MQ water and incubated in 8% acetic acid at room temperature (RT) for 1 min. The samples were analyzed with epifluorescence microscopy under green-light excitation (Leica Aristoplan; Leica Biosystems GmbH, Wetzlar, Germany).

### Gas chromatography.

The PHA content and composition in the PHA biopolymers (PHB, polyhydroxyvalerate (PHV) and polyhydroxyoctanoate (PHO) as standards as well as from the biomasses of *Paracoccus* sp. 392 and *Halomonas* sp. 363 were determined with gas chromatography-mass spectrometry (GC-MS) as described below. The cells were collected (13,000 × *g*, 3 min, 4°C) from the N-limited 1-wk and N-replete 1-wk treatments, washed with N-limiting growth medium, and freeze-dried for 20 h (100 Pa, +3.5 Pa final dry for 2 h). In all, 10 mg of lyophilized cells (or 1 mg of isolated PHAs, respectively) was subjected to methanolysis, which was done in a mixture of 2 ml high-performance liquid chromatography (HPLC)-grade chloroform and 2 ml methanol containing 15% (vol/vol) sulfuric acid, as suggested previously (70, 71). The samples were diluted 50-fold with n-hexane of HPLC grade. The initial structural assignments of the methyl esters obtained were based on their retention times compared with those of authentic standards of practical (PA) grade, including methyl (S)-(R)-3-hydroxybutyrate 98% from Alfa Aesar (Thermo Fisher Scientific, Haverhill, MA, USA), (−)-methyl-(R)-3-hydroxyvalerate, 98% from Sigma-Aldrich (now Millipore Sigma, Burlington, MA, USA), methyl-3-hydroxyhexanoate from Sigma-Aldrich, and methyl-(S)-3-hydroxyoctanoate from Key Organics Ltd., Camelford, Cornwall, UK (ordered through Sigma-Aldrich).

For each analysis, we applied a hexane blank for monitoring the thermocycle and purities of the column. The authentic structures of the monomers were determined by GC-MS, using an Agilent Technologies LDA UK Ltd. (Stockport, Cheshire, UK) instrument with a capillary column of type Agilent HP-5MS UI 30 m, 0.25 mm, and the carrier gas (99.9999% purity helium at a constant flow of 1.2 ml/min). The temperature program was modified with an initial temperature of 40°C with a hold of 2 min, followed by a ramp of 20°C/min to 140°C, a second ramp of 40°C/min to 300°C, and then a hold at 300°C for 3 min, giving a total run time of 14 min. For the detector settings, a transfer line temperature of 250°C and mass-to-charge ratio (*m/z*) scanning range of 50 to 300 were applied.

### DNA extraction, library preparation, and sequencing.

DNA was extracted from 1 ml of ZoBell growth medium with a DNeasy UltraClean microbial kit (Qiagen, Hilden, Germany) and stored at –80°C. Whole-genome large-insert (16 kbp for *Paracoccus* sp. 392; 14 kbp for *Halomonas* sp. 363) PacBio libraries for the RSII instrument were prepared, using a DNA Template Prep Kit 2.0 and DNA/Polymerase Binding Kit P6 according to the manufacturer’s protocol. Both samples were sequenced individually in a single-molecule, real-time (SMRT) cell. Dual-indexed paired-end genomic DNA (gDNA) libraries were prepared according to the Illumina Nextera DNA library prep guide (Illumina Inc., San Diego, CA, USA), except that half of the Tagment DNA enzyme 1 (TDE1) was used per reaction. An Illumina NextSeq 500 instrument was used to sequence the DNA fragments in a paired-end manner (170 + 132 bp).

### RNA extraction, cDNA translation, and library preparation and sequencing.

RNA was extracted using the cetyltrimethylammonium bromide-polyethylene glycol (CTAB-Peg) DNA/RNA extraction protocol (72), after which the RNA was purified with an AllPrep DNA/RNA kit (Qiagen, Hilden, Germany). The libraries were prepared according to the manufacturer’s instructions with a NEBNext Ultra II RNA library prep kit for Illumina (number E7770, New England BioLabs, Inc.), using NEBNext Multiplex Oligos for Illumina 96 index primers (number E6609S, New England BioLabs, Inc.) and NEBNext sample purification beads (number E7767S, New England BioLabs, Inc.). Paired-end (75 + 75) sequencing was performed on an Illumina Nextseq 500 instrument.

### Bioinformatics pipeline.

**(i) Genomes.** The PacBio reads were assembled using the hierarchical genome assembly process 3 (HGAP3) implemented in smartportal 2.3.0 (Pacific Biosciences, Menlo Park, CA, USA), using default parameters. The sequences obtained were manually inspected and circularized, using the GAP4 Staden package (73). Chromosomal DNA sequencing was set to start from the *dnaA* gene. The Illumina short reads were first quality checked with FastQC (74) and then filtered using Cutadapt (v. 1.14) (75) with the following three criteria: (i) adapter sequence removal; (ii) removal of low-quality bases from the 3′ end of the read (-q 25); and (iii) minimum read length (-m 50) set to 50 bp. The filtered Illumina short reads were mapped against the circularized sequences with bwa mem (v. 0.7.17) (76), then sorted and indexed with SAMtools (v. 1.7) (77). Reads that did not map to the reference sequences given were selected and assembled separately with spades (v. 3.11.1) (78), using the -careful option. Sequences from the spades assembly were circularized in GAP4. Finally, all sequences were polished using pilon (v. 1.16) (79).

The sequences were annotated against the Kyoto Encyclopedia of Genes and Genomes (KEGG) (release 86, April 2018) (80) with KEGG-tools2.0 (81), PROKKA (1.13) (39), and RAST with default parameters (2.0) (38).

**(ii) Transcriptomes.** The quality of the raw reads was analyzed with FastQC (74). The primers were removed with Cutadapt (v. 1.10 with Python 2.7.3) (75, 82), using a quality score of 20 and minimum length of 30. The complementary DNA (cDNA) was annotated against PROKKA (1.13) (39), and the trimmed reads were mapped against the PROKKA-annotated genes (ffn-file) with Bowtie2 (v.1.2.2) (83) and sorted and indexed with SAMtools (v. 1.4) (77).

### Statistics.

Differences between the treatments for selected genes were tested with one-way analysis of variance (ANOVA, function “aov” in R-core package R4.0.2) (84). Variance of homogeneity (*P* > 0.05) was tested with Levene’s test (package “car” in R4.0.2) and normality (*P* > 0.05) with Shapiro-Wilk normality test (84). Tests were done only for *Halomonas* sp. 363, since one of the *Paracoccus* sp. 392 replicates failed to grow and thus made the statistical tests unreliable.

For further analyses, the rRNA-associated transcripts were removed and abundances were normalized against the single-copy gene *rpoB*. Data cleaning was done using the package tidyverse (1.3.0) (85). The graphics were done in R4.0.2 (84) using ggplot2 (3.3.2) (86) and pheatmap (1.0.12) (87).

### Data and code availability.

The data reported in this article is available in Tables S1 and S2 in the supplemental material. The raw RNA-seq fastq sequence data files are deposited in the European Nucleotide Archive (ENA) study PRJEB41946 under accession numbers ERS5465044 (SAMEA7708542) and ERS5465113 (SAMEA7708611), and closed genomes of *Halomonas* sp. 363 and *Paracoccus* sp. 392 are found under accession numbers ERS5472646 (SAMEA7725270) and ERS5472645 (SAMEA7725269), respectively.

All scripts for processing RNA-seq data are available in the supplemental material. PHA_experiment_bioinformatics.html and R-scripts are at https://github.com/elxerone/PHA_experiments.
