# Supplementary material for: Sea-Ice Bacteria Halomonas sp. Strain 363 and Paracoccus sp. Strain 392 Produce Multiple Types of Poly-3-Hydroxyalkaonoic Acid (PHA) Storage Polymers at Low Temperature
Source: Appl Environ Microbiol. 2021 Aug 11;87(17):e00929-21. doi: 10.1128/AEM.00929-21 (PMC8357295; doi:10.1128/AEM.00929-21)
Supplement: Supplemental file 1 — Figures S1 to S7, table legends, Document S1, bioinformatics pipeline. Download AEM.00929-21-s0001.pdf, PDF file, 2.2 MB [file aem.00929-21-s0001.pdf]

Supplementary figures

Extremophilic sea-ice bacteria, *Halomonas* sp. 363 and *Paracoccus* sp. 392, produce multiple types of poly-3-hydroxyalkaonoic acid (PHA) storage polymers at low temperature

*Eronen-Rasimus, E.[a,b]#, Hultman, J., Hai, T., Pessi, I.S., Collins, E., Wright, S., Laine, P., Viitamäki, S., Lyra, C., Thomas, D.N., Golyshin, P., Luhtanen, A.-M., Kuosa, H. and Kaartokallio, H.

#Address correspondence to eeva.eronen-rasimus@helsinki.fi: +358294159317 eeva.eronen-rasimus@syke.fi: +358 46 9221008

[a] *University of Helsinki, Faculty of Agriculture and Forestry, Department of Microbiology, Helsinki, Finland, PO BOX 56 (Viikinkaari 9), FI-00014 University of Helsinki, Finland*

[b]*Finnish Environment Institute (SYKE), Marine Research Centre, Helsinki, Finland*

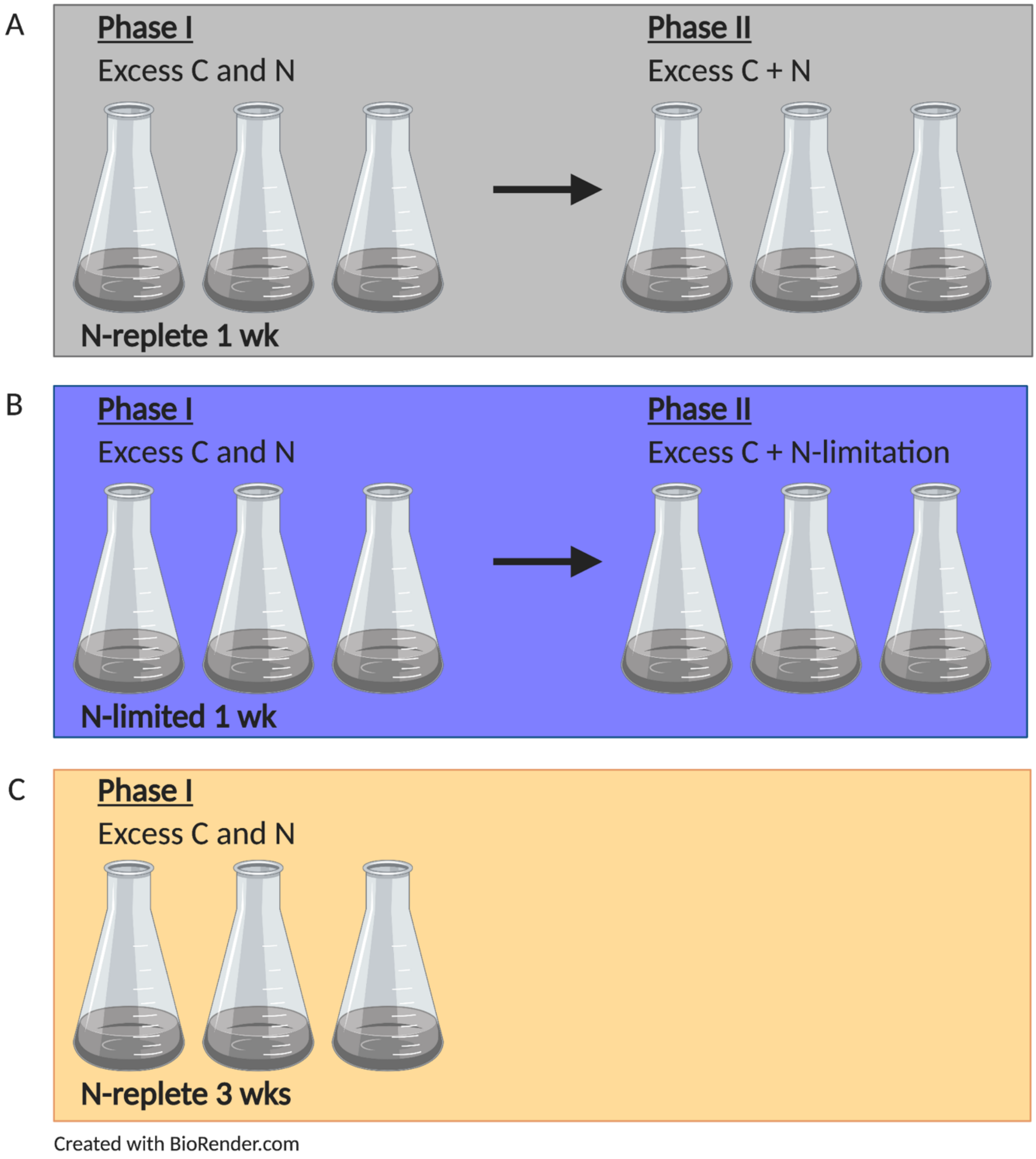

Figure S1. Experimental set up for the shaker flask batch-culture experiments for *Halomonas* sp. 363 and *Paracoccus* sp. 392 cultured with glucose and gluconate in (A) N-replete 1 wk treatment, (B) N-limited 1 wk treatment, (C) N-replete 3 wks treatment (*Halomonas* sp. 363 only). The samples were collected at the end of Phase I (Day1) and subsequently transferred to the new media for Phase II. The samples were collected daily for 4 days from the N-limited 1 wk and N-replete 1 wk treatments (Days 2–5) and once a week in the N-replete 3 wks treatment for 3 weeks (Days 5, 12 and 19).

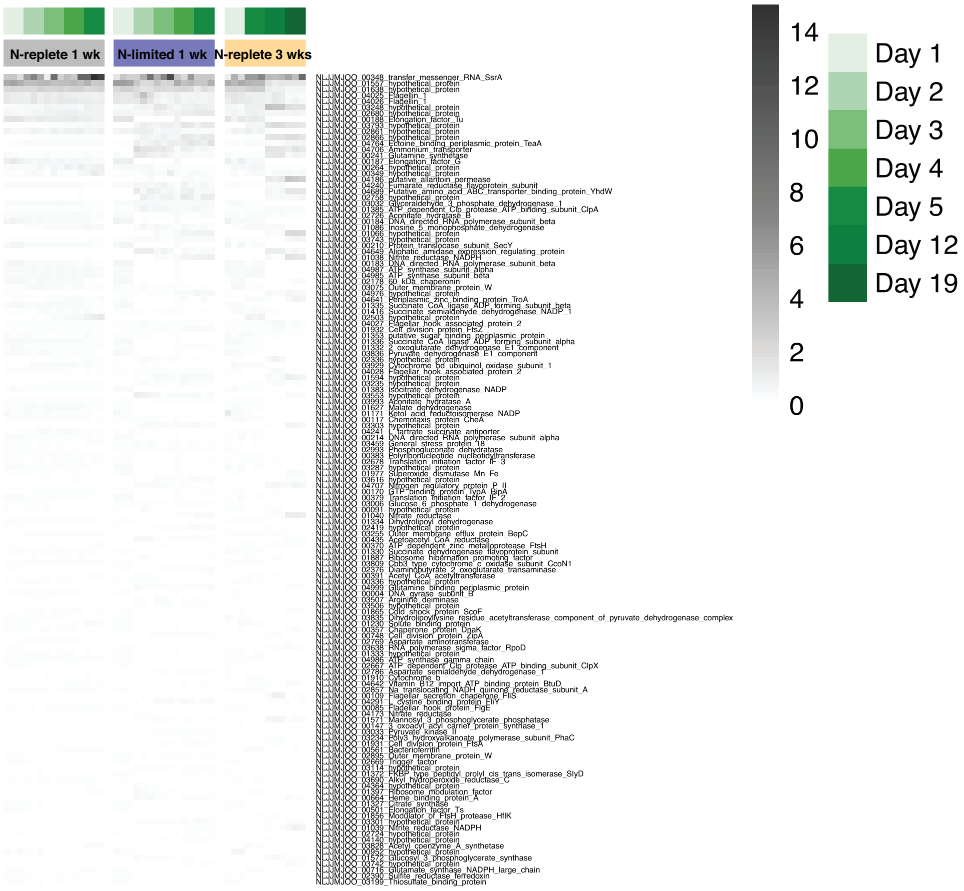

Figure S2. Relative percentage (> 0.1%) of actively transcribed, *rpoB*-normalized PROKKA-annotated transcripts after rRNA gene removal of *Halomonas* sp. 363.

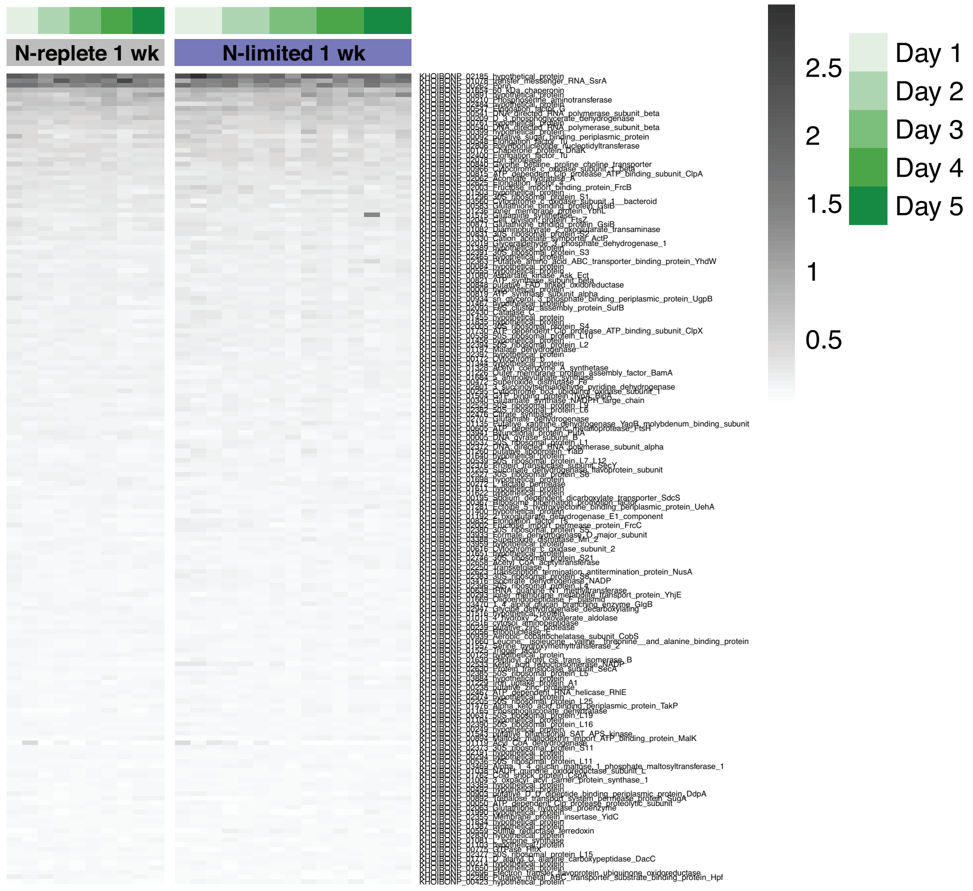

Figure S3. Relative percentage (> 0.1%) of actively transcribed PROKKA annotated transcripts in *Paracoccus* sp. 392. The sequences were normalized against *rpoB*.

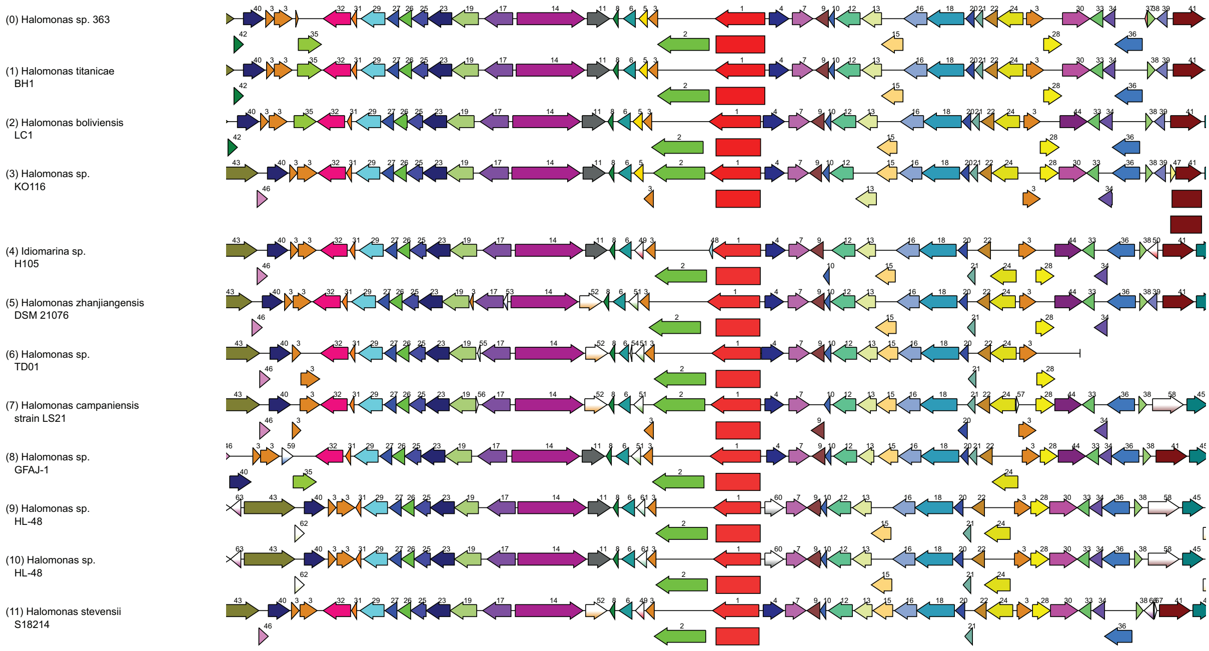

Figure S4. *Halomonas* sp. 363 *phaC* gene aligned with genes from other *Halomonas* strains from PATRIC (3.6.7, 26.11.2020) (Davis et al., 2015).

A *Halomonas* sp. 363 Fatty Acid Biosynthesis (FAB) genes

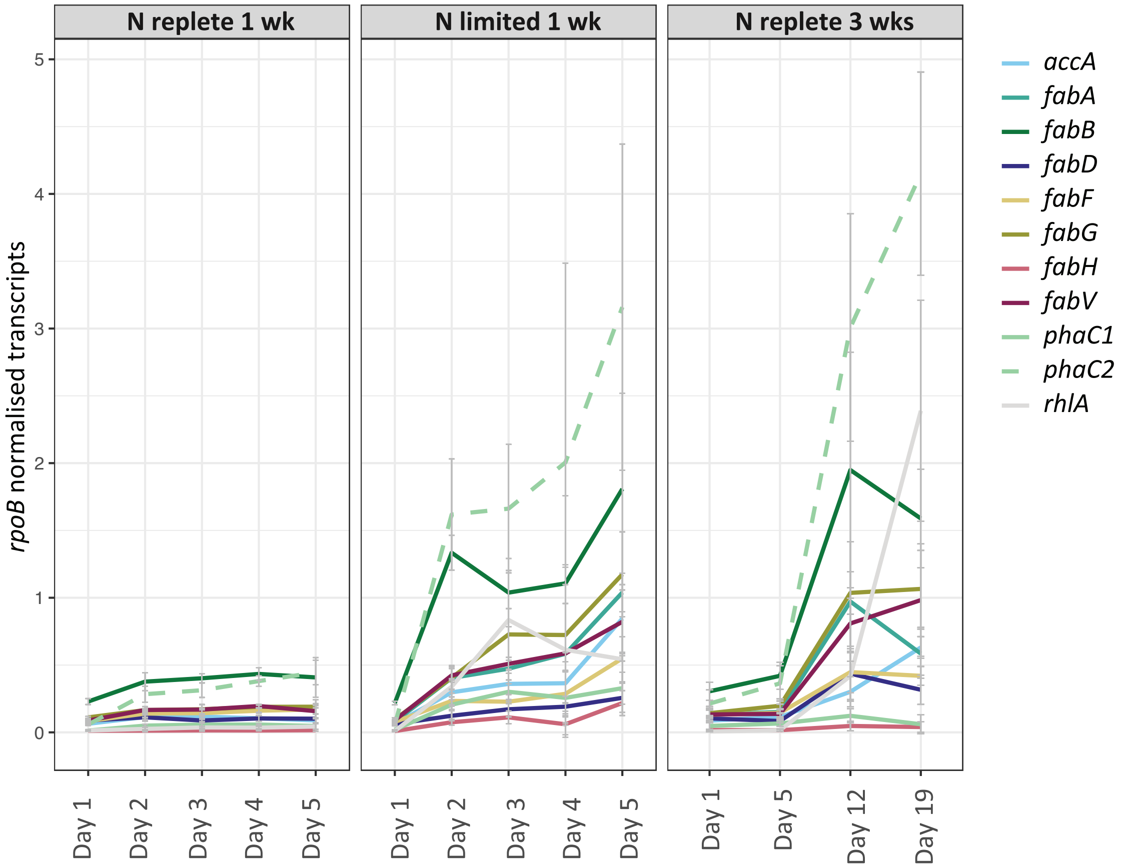

B *Halomonas* sp. 363 Fatty Acid Degradation (FAD) genes

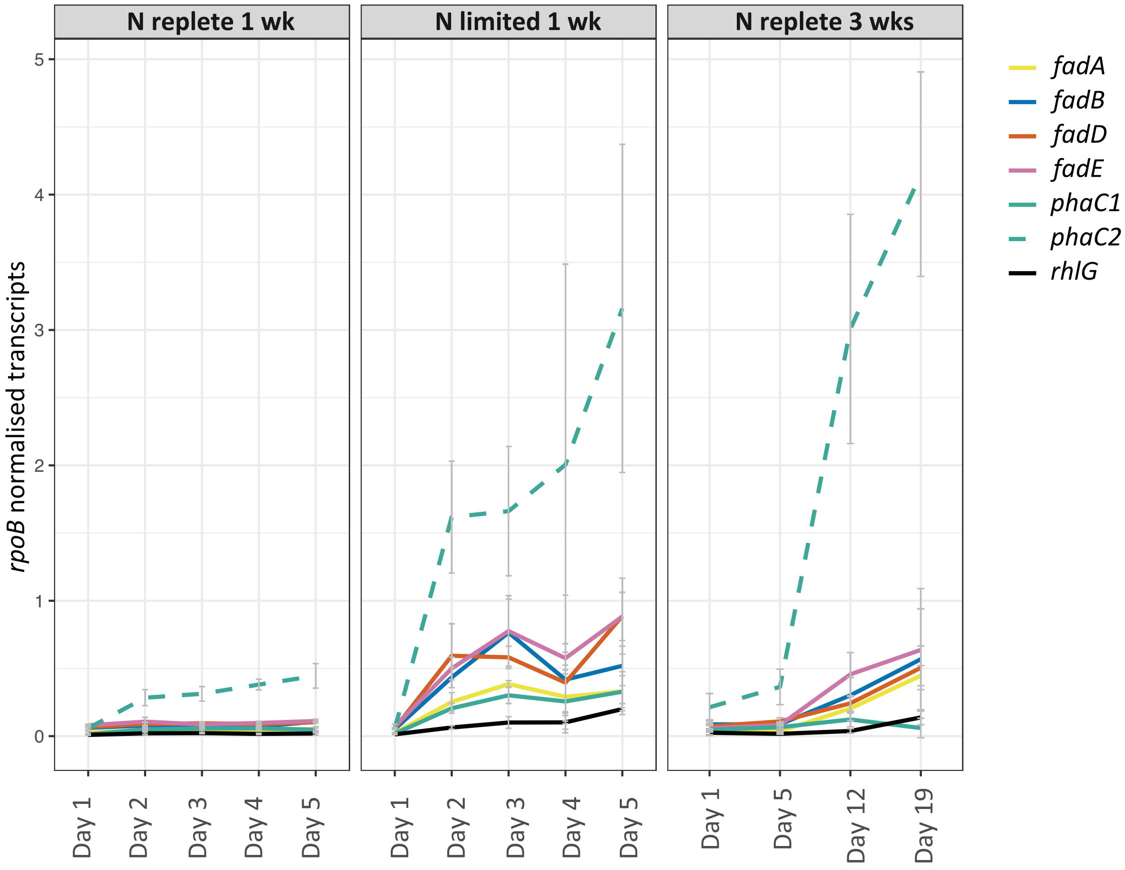

Figure S5. Actively transcribed PROKKA annotated transcripts in *Halomonas* sp. 363 associated with A.) Fatty-Acid Biosynthesis and B.) Fatty-Acid Biodegradation. The sequences were normalized against *rpoB*.

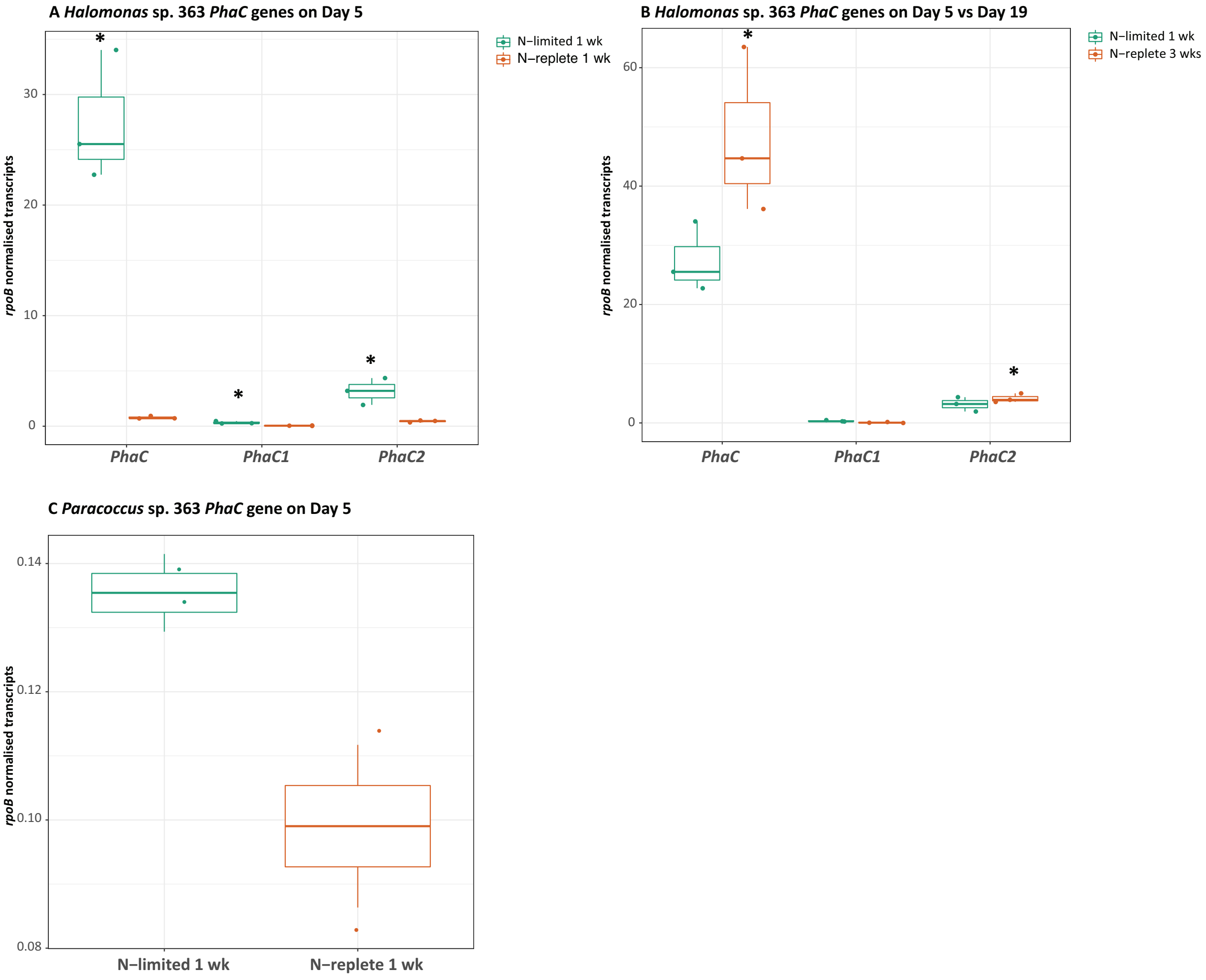

Figure S6. *PhaC* gene transcripts between A.) N-limited 1 wk and N-replete 1 wk on Day 5 in *Halomonas* sp. 363 B.) N-limited 1 wk on Day 5 and N-replete 3 wks on Day 19 in *Halomonas* sp. 363 and C.) N-limited 1 wk and N-replete 1 wk on Day 5 in *Paracoccus* sp. 392. Asterisk (*) denotes statistically significant differences ($P < 0.05$). Statistical test for *Paracoccus* sp. 392 was not done since one of the three replicates was compromised. Note different scales!

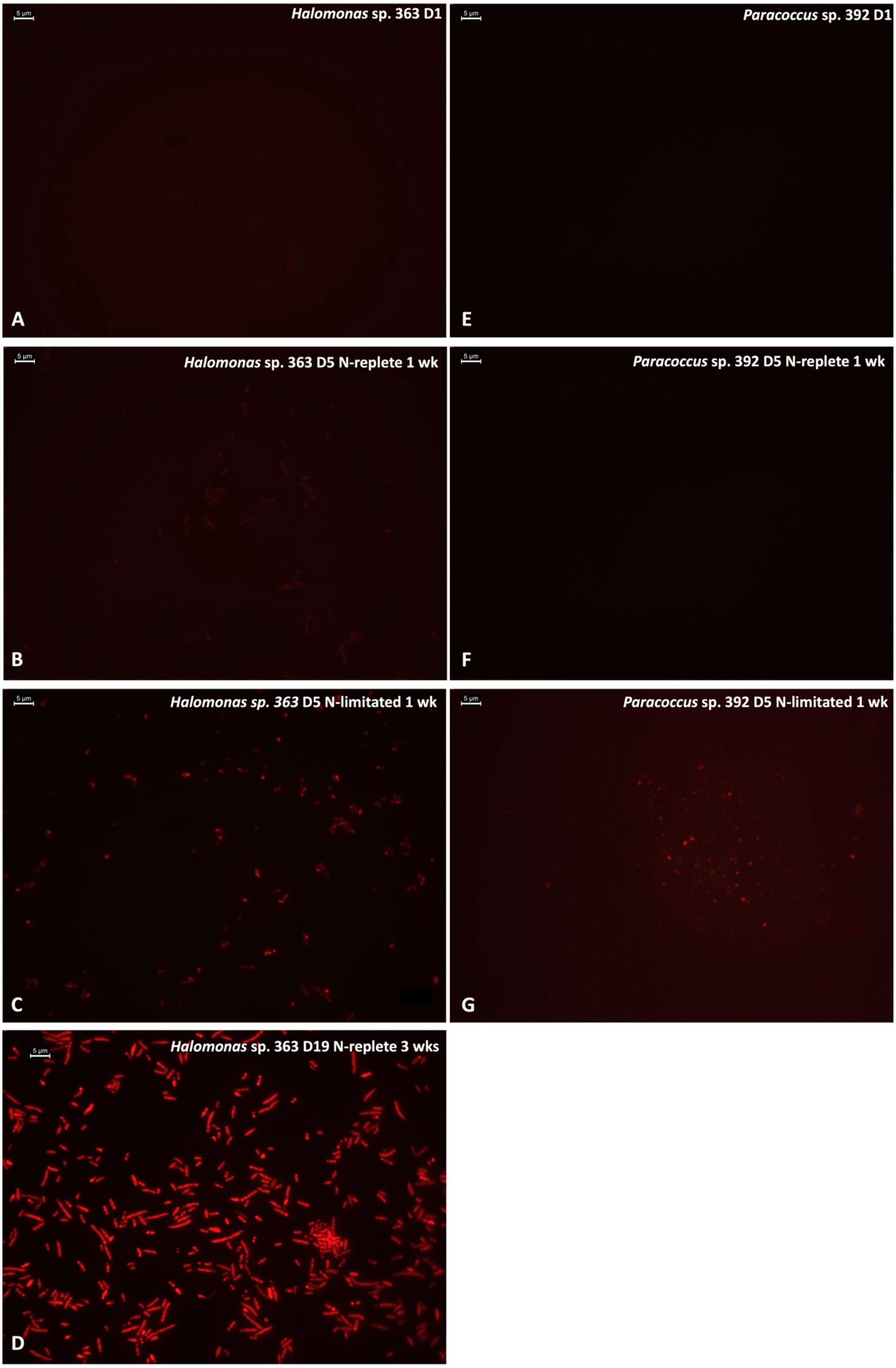
Halomonas sp. 363 D1
A
Paracoccus sp. 392 D1
E
Halomonas sp. 363 D5 N-replete 1 wk
B
Paracoccus sp. 392 D5 N-replete 1 wk
F
Halomonas sp. 363 D5 N-limitated 1 wk
C
Paracoccus sp. 392 D5 N-limitated 1 wk
G
Halomonas sp. 363 D19 N-replete 3 wks
D
5 µm

Figure S7. Epifluorescence micrographs of Nile-blue A-stained 206 Halomonas sp. 363 and *Paracoccus* sp. 392 cells from shaker flask batch-culture experiments in (A–D) *Halomonas* 363 and (E-F) *Paracoccus* 392. The poly-3-hydroxyalkanoic acid (PHA) granules in the cell are seen in red.

Table S1. PROKKA- (1.13) (Seemann, 2014), RAST- (2.0) (Aziz et al., 2008) and KEGG- (86, April 2018) (Kanehisa and Goto, 2000) annotated *Halomonas* sp. 363 genomes.

Table S2. PROKKA- (1.13) (Seemann, 2014), RAST- (2.0) (Aziz et al., 2008) and KEGG- (86, April 2018) (Kanehisa and Goto, 2000) annotated *Paracoccus* sp. 392 genomes.

Table S3. PROKKA- (1.13) (Seemann, 2014), RAST- (2.0) (Aziz et al., 2008) and KEGG- (86, April 2018) (Kanehisa and Goto, 2000) annotated *Halomonas* sp. 363 transcripts.

Table S4. PROKKA- (1.13) (Seemann, 2014), RAST- (2.0) (Aziz et al., 2008) and KEGG- (86, April 2018) (Kanehisa and Goto, 2000) annotated *Paracoccus* sp. 392 transcripts.

Table S5. PROKKA- (1.13) (Seemann, 2014), RAST- (2.0) (Aziz et al., 2008) and KEGG- (86, April 2018) (Kanehisa and Goto, 2000) annotated genes associated with PHA-production.

1. Davis JJ, Wattam AR, Aziz RK, Brettin T, Butler R, Butler RM, Chlenski P, Conrad N, Dickerman A, Dietrich EM, Gabbard JL, Gerdes S, Guard A, Kenyon RW, Machi D, Mao C, Murphy-Olson D, Nguyen M, Nordberg EK, Olsen GJ, Olson RD, Overbeek JC, Overbeek R, Parrello B, Pusch GD, Shukla M, Thomas C, VanOeffelen M, Vonstein V, Warren AS, Xia F, Xie D, Yoo H, Stevens R. 2020. The PATRIC Bioinformatics Resource Center: expanding data and analysis capabilities. Nucleic Acids Res. Jan 8: 48(D1):D606-D612. PMID: 31667520. PMCID: PMC7145515.

2. Aziz RK, Bartels D, Best AA, DeJongh M, Disz T, Edwards RA, Formsma K, Gerdes S, Glass EM, Kubal M, Meyer F, Olsen GJ, Olson R, Osterman AL, Overbeek RA, McNeil LK, Paarmann D, Paczian T, Parrello B, Pusch GD, Reich C, Stevens R, Vassieva O, Vonstein V, Wilke A, Zagnitko O. 2008. The RAST Server: rapid annotations using subsystems technology. BMC genomics: 9: 1–15.
3. Seemann T. 2014. Prokka: Rapid Prokaryotic Genome Annotation, Bioinformatics 15: 2068–9.
4. Kanehisa M, Goto S. 2000 KEGG: Kyoto Encyclopedia of Genes and Genomes. Nucleic Acids Res 28: 27–30.

Bioinformatics pipeline PHA experiment

Eeva Eronen-Rasimus

10/28/2020

Genomes

Genome files:
- *Halomonas*: Sample_363_chr_31102019.Final.fasta
- *Paracoccus*: NEW10.392.05122019.fasta

Genomes were annotated with Prokka 1.13 (Seemann T., 2014), RAST (Aziz et al., 2008) and KEGG (Kanehisa and Koto, 2000) with KEGG-tools2.0 (Pessi, 2019)

prokka Sample_363_chr_31102019.Final.fasta –outdir PROKKA_363_closed

Genome annotations available:
ST1_Genomes_Halomonas.xlsm Sheets: Genomes_Halomonas_PROKKA, Genomes_Halomonas_RAST, Genomes_Halomonas_KEGG

ST2_Genomes_Paracoccus.xlsm Sheets: Genomes_Paracoccus_PROKKA, Genomes_Paracoccus_RAST, Genomes_Paracoccus_KEGG

cDNA Mapping

Halomonas example

Trimming

Quality of sequences was checked with Fastqc (v. 0.11.8, Andrews, 2010) and adapters were removed with Cutadapt (v. 1.10 with Python 2.7.3, Martin, 2011,Van Rossum and Drake, 1995)

```
# set task to be processed
i=$(sed -n "$SLURM_ARRAY_TASK_ID"p sample_names_PHA.txt)

# run the analysis command
cutadapt -a GATCGGAAGAGCACACGTCTGAACTCCAGTC -A GATCGGAAGAGCGTCGTGTAGGGAAAGAGTGT -m 30 -q 20 \
-o ../TRIMMED/$i"_R1_trimmed.fastq" -p ../TRIMMED/$i"_R2_trimmed.fastq" \
*$i*_R1*.fastq *$i*_R2*.fastq > ../TRIMMED/$i"_trim.log"
```

Mapping

cDNA reads were mapped against Prokka annotated CDSs (ffn-files).

Replace white space with "_" to tag annotation along

```
sed 's/ /_/g' PROKKA_HAL_closed.ffn > PROKKA_HAL_closed2.ffn
```

Make bowtie2 (Bowtie2 (v.1.2.2, Langmead et al, 2012) indexed database

```
bowtie2-build PROKKA_HAL_closed2.ffn 363_closed_ffn_db
```

Map the trimmed reads

```
i=$(sed -n "$SLURM_ARRAY_TASK_ID"p ../../TRIMMED/sample_names_HAL.txt)
bowtie2 -x 363_closed_fnn_db \
-1 ../../TRIMMED/$i"_R1_trimmed.fastq" -2 ../../TRIMMED/$i"_R2_trimmed.fastq" \
-S "spades_map_closed"$i.sam
```

Make a table with SAMtools (v 1.4, Li et al., 2009)
(Script for get_count_table.py available: https://github.com/edamame-course/Metagenome/blob/master/get_count_table.py)

```
samtools view -F 4 -bS "spades_map_closed"$i.sam > "spades_map_closed"$i.bam
samtools sort "spades_map_closed"$i.bam -o "spades_map_closed"$i.bam.sorted
samtools index "spades_map_closed"$i.bam.sorted
samtools idxstats "spades_map_closed"$i.bam.sorted > IDX_HAL/$i"closed.idxstats.txt"
python get_count_table.py H*.idxstats.txt > count_closed_HAL.txt
```

Tables available in:
ST3_cDNA_Halomonas_PROKKA.xlsm
ST4_cDNA_Paracoccus_PROKKA.xlsm

References

Andrews, S. (2010). FastQC: A Quality Control Tool for High Throughput Sequence Data [Online]. Available online at: http://www.bioinformatics.babraham.ac.uk/projects/fastqc/
Aziz, R. K., Bartels, D., Best, A. A., DeJongh, M., Disz, T., Edwards, R. A., … & Meyer, F. (2008). The RAST Server: rapid annotations using subsystems technology. BMC genomics, 9(1), 1-15.
Kanehisa, M. and Goto, S.; KEGG: Kyoto Encyclopedia of Genes and Genomes. Nucleic Acids Res. 28, 27-30 (2000). [pubmed] [doi]
Langmead B, Salzberg S. Fast gapped-read alignment with Bowtie 2. Nature Methods. 2012, 9:357-359.
Li, H., Handsaker, B., Wysoker, A., Fennell, T., Ruan, J., Homer, N., … & Durbin, R. (2009). The sequence alignment/map format and SAMtools. Bioinformatics, 25: 2078-2079.
Martin, M. (2011). Cutadapt removes adapter sequences from high-throughput sequencing reads. EMBnet. journal, 17(1), 10-12.
Seemann T., Prokka: rapid prokaryotic genome annotation. Bioinformatics. 2014 Jul 15;30(14):2068-9. doi: 10.1093/bioinformatics/btu153. Epub 2014 Mar 18. PMID: 24642063
Pessi IS. 2019. KEGG-tools v2.0: A tool to parse the results of BLAST/DIAMOND similarity searches made against the KEGG GENES prokaryotes database. GitHub Repository. DOI: https://doi.org/10.5281/zenodo.1243746
Van Rossum, G., & Drake Jr, F. L. (1995). Python reference manual. Centrum voor Wiskunde en Informatica Amsterdam.
